# A Case Report of Ogilvie’s Syndrome in a 58-year-old Quadriplegic

**DOI:** 10.21980/J82922

**Published:** 2020-10-15

**Authors:** Rosie Kumar, Brett Cowan, Daniel Quesada, Sage Wexner

**Affiliations:** * Kern Medical Center, Department of Emergency Medicine, Bakersfield, CA

## Abstract

Ogilvie’s syndrome (acute colonic pseudo-obstruction) is a rare disorder characterized by an acute dilation of the colon measuring greater than 10 centimeters. Common symptoms associated with Ogilvie’s include abdominal distension, abdominal pain, nausea, vomiting, constipation, and diarrhea. This report presents an uncommon case of a quadriplegic presenting to the emergency department with complaints of abdominal distention. Computed tomography and radiographic imaging studies were consistent with Ogilvie’s syndrome. The patient was admitted to the hospital and started on stool softeners and bowel rest with resolution of symptoms.

**Topics:**

Ogilvie’s syndrome, quadriplegic, abdominal distension, colonic pseudo-obstruction.

**Figure:**
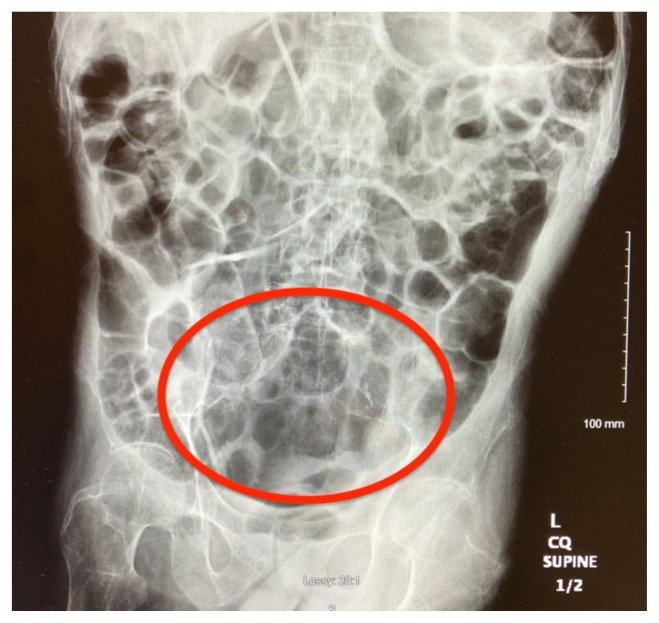

**Figure:**
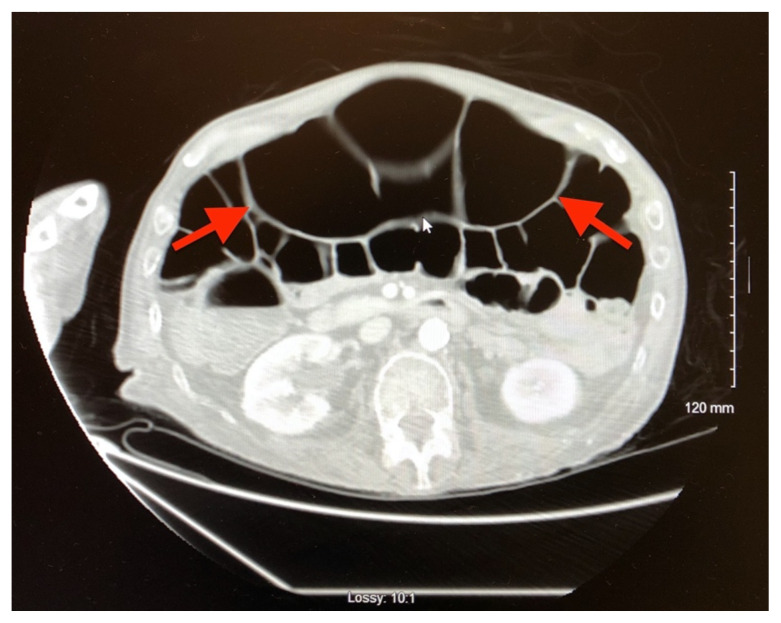

## Introduction

The distention caused by Ogilvie’s syndrome is not due to a mechanical obstruction and typically involves the cecum and right hemicolon; however, extension can occasionally extend to the rectum.^1^ This can result in the blood supply being compromised and lead to perforation, peritonitis, and gangrene.^4^ Those at risk for developing this syndrome are patients who are hospitalized or institutionalized, over the age of 60, have a severe illness, such as nonoperative trauma, infection, or cardiac disease, or/and who have undergone surgeries, such as a cesarean section and hip surgery.^1^,^2^ Neurological conditions including Parkinson’s disease, multiple sclerosis, Alzheimer disease, and a spinal cord injury may also contribute to the development of Ogilvie’s.^4^ Additional risk factors include obesity, and cumulative doses of prednisone, or mycophenolate mofetil^1^. This case is important because it demonstrates how conservative treatment as per guidelines can be effective in treating this condition.

## Presenting concerns and clinical findings

A 58-year-old male with a past medical history significant for quadriplegia status post gunshot wound and previous exploratory laparotomy in 1980 with neurogenic bladder and indwelling Foley catheter, presented to the emergency department after endorsing worsening abdominal distention for the past two days. The patient stated he was in his usual state of health, eating normally, passing flatus, last bowel movement the day prior to presentation, until he noticed that his abdomen was getting increasingly swollen. He denied any associated nausea or vomiting, seizures, falls, worsening mental status, or any other acute complaints. A suspicion of possible small bowel obstruction, large bowel obstruction, ischemic colitis, toxic megacolon, and malignancy prompted radiographic investigation.

## Significant findings

Plain radiograph of the patient’s abdomen revealed a gaseous distention of the colon. This is demonstrated as noted in the abdominal x-ray as gaseous distention, most notably in the large bowel (arrows) including the rectal region (large circle). Follow up computed tomography (CT) scan affirmed severe pancolonic gaseous distention measuring up to 11.2 cm, compatible with colonic pseudo-obstruction as noted by the large red arrows. No anatomical lesion or mechanical obstruction was observed, as well as no evidence of malignancy or other acute process.

## Patient Course

This patient was placed on nil per os (NPO), except for medications, had a nasogastric tube placed and set to suction, and was given stool softeners including polyethylene glycol (Miralax) 17g, magnesium hydroxide (Milk of Magnesia) 10mL, and docusate-senna 50mg-8.6mg each given multiple times over a course of 5 days. The decision was made to not start the patient on treatment with Neostigmine because colonic distension did not exceed 12cm. The patient improved with conservative management. Upon discharge the patient was having normal bowel movements, resolution of abdominal distention, and was tolerating oral intake normally.

## Discussion

The exact mechanism leading to Ogilvie’s syndrome is unknown^1^. However, it is believed that impairment of the autonomic nervous system from trauma, spinal anesthesia, and pharmacologic agents, as well as interruption of the parasympathetic fibers from S2 to S4, can lead to distention of the colon.^1^ The increase in the colonic diameter can increase the tension on the colonic wall^1^. The primary clinical manifestations in patients with this syndrome is abdominal distension which typically occurs gradually over three to seven days, but can also develop rapidly within 24 to 48 hours.^1^ Patients may also present with abdominal pain, nausea, vomiting, constipation, and diarrhea.^1^

Laboratory tests typically include a complete blood count, serum lactate levels and electrolytes. Tests may reveal leukocytosis, which is usually due to the patient’s underlying disease or a sign of impending colon perforation.^1^ Additionally, metabolic abnormalities such as hypokalemia, hypocalcemia, and hypomagnesemia can be present.^4^ Hypothyroidism should also be screened because, in rare cases, this condition can be associated with colonic distension and is eminently treatable.^1^ Abdominal CT scan or x-ray revealing colonic dilatation is used to diagnose a patient with Ogilvie’s syndrome.^1^

Initial treatment includes conservative measures such as nasogastric decompression, bowel rest, correction of electrolytes, and cessation of any medications, such as opioids, which could exacerbate the condition.^3^ If condition does not improve within 24–48 hours, some experts believe there is evidence to treat Ogilvie’s syndrome with neostigmine, an anticholinesterase agent that allows increased synaptic levels of acetylcholine to counteract the sympathetic-parasympathetic imbalance associated with dilatation of the colon.^3^ Alternative forms of treatment for patients who fail medical therapy include endoscopic decompression and cecostomy.^3^ The main takeaway from this case is the response the patient had to conservative treatment as described above, and that with distension less than 12 cm, this case supports conservative treatment alone without the necessity of using neostigmine. Possible limitations include the application of this case to cases with a larger degree of colonic distension, as well as application to patients whose predisposing risk factors are different from this patient’s. While these are important aspects to consider, treatment in this case was consistent with current recommendations and resulted in positive outcomes.

## Supplementary Information








