# Supplementary figures and images for: A Case Report of Ogilvie’s Syndrome in a 58-year-old Quadriplegic
Source: J Educ Teach Emerg Med. 2020 Oct 15;5(4):V19–21. doi: 10.21980/J82922 (PMC10332525; doi:10.21980/J82922)

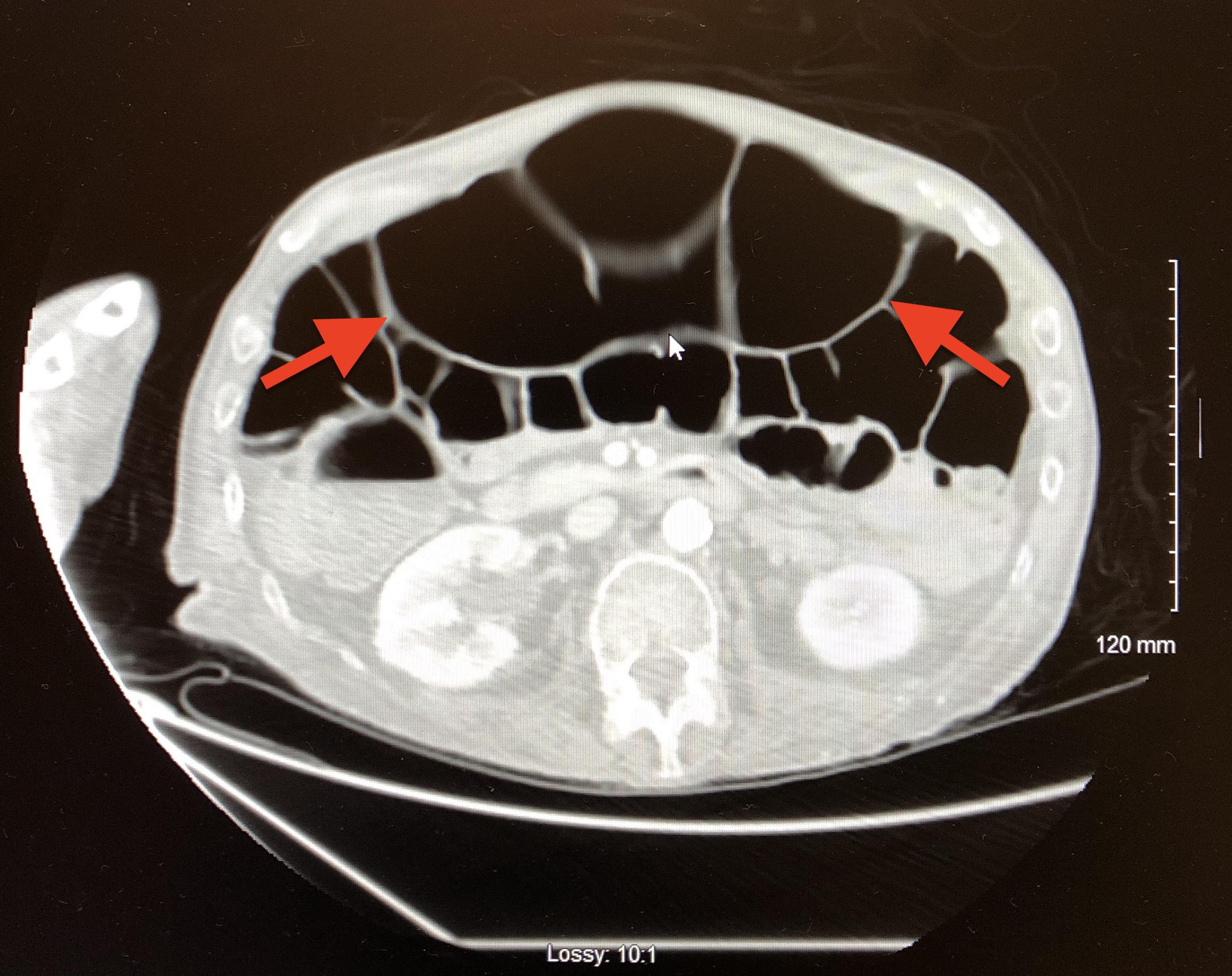

Supplement: Supplementary file 1 [file jetem-5-4-v19-supp1.jpg]

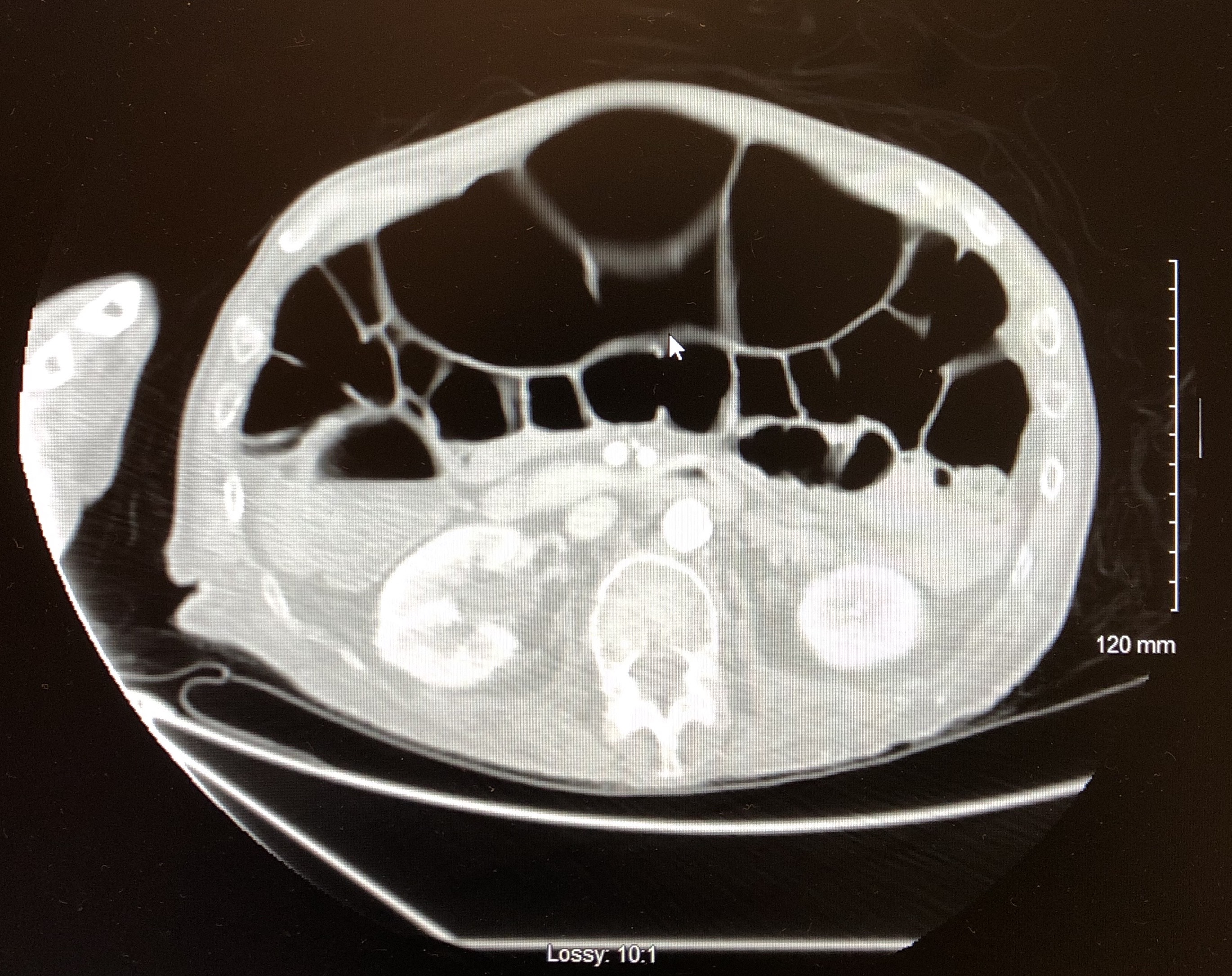

Supplement: Supplementary file 2 [file jetem-5-4-v19-supp2.jpg]

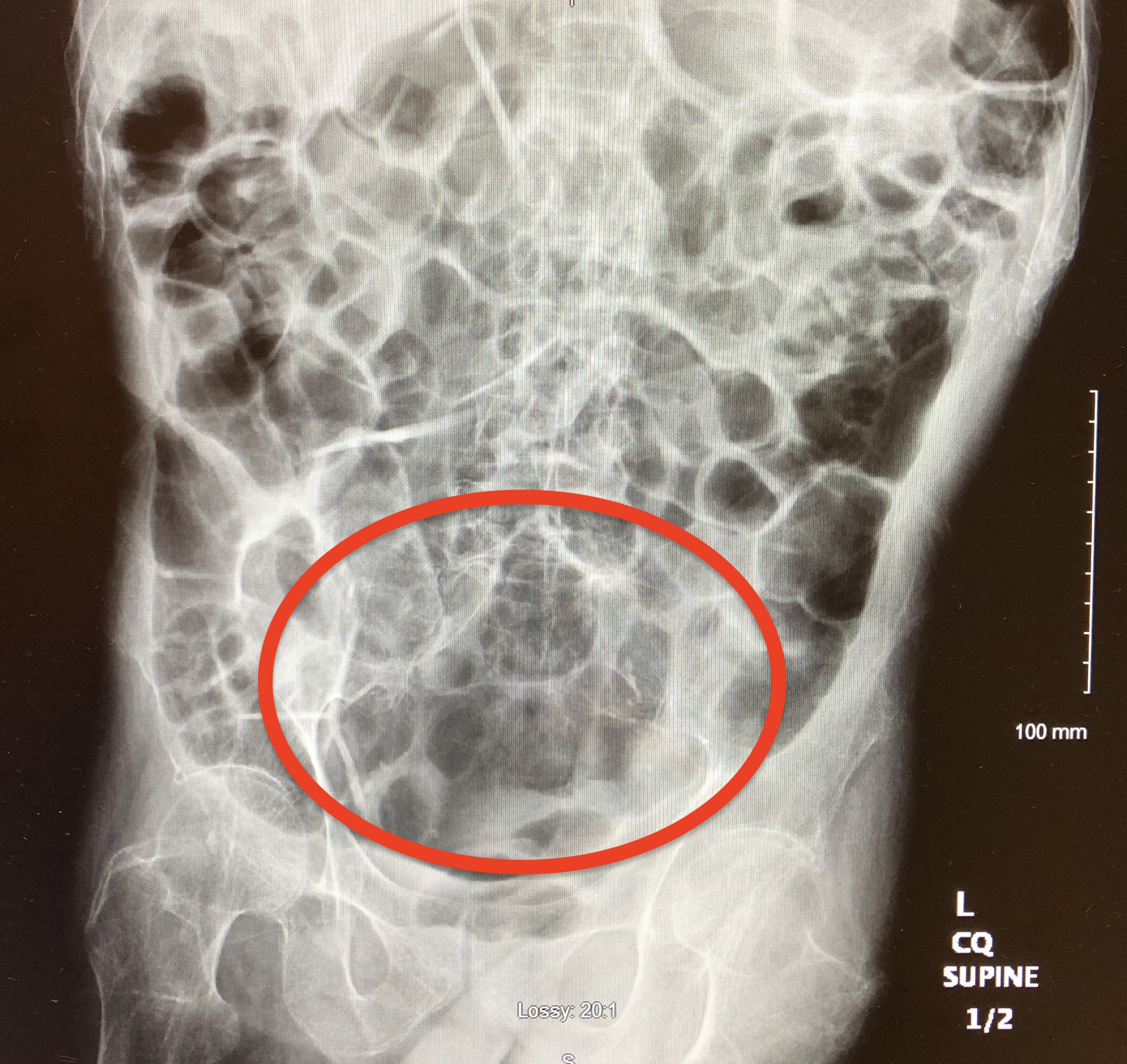

Supplement: Supplementary file 3 [file jetem-5-4-v19-supp3.jpg]

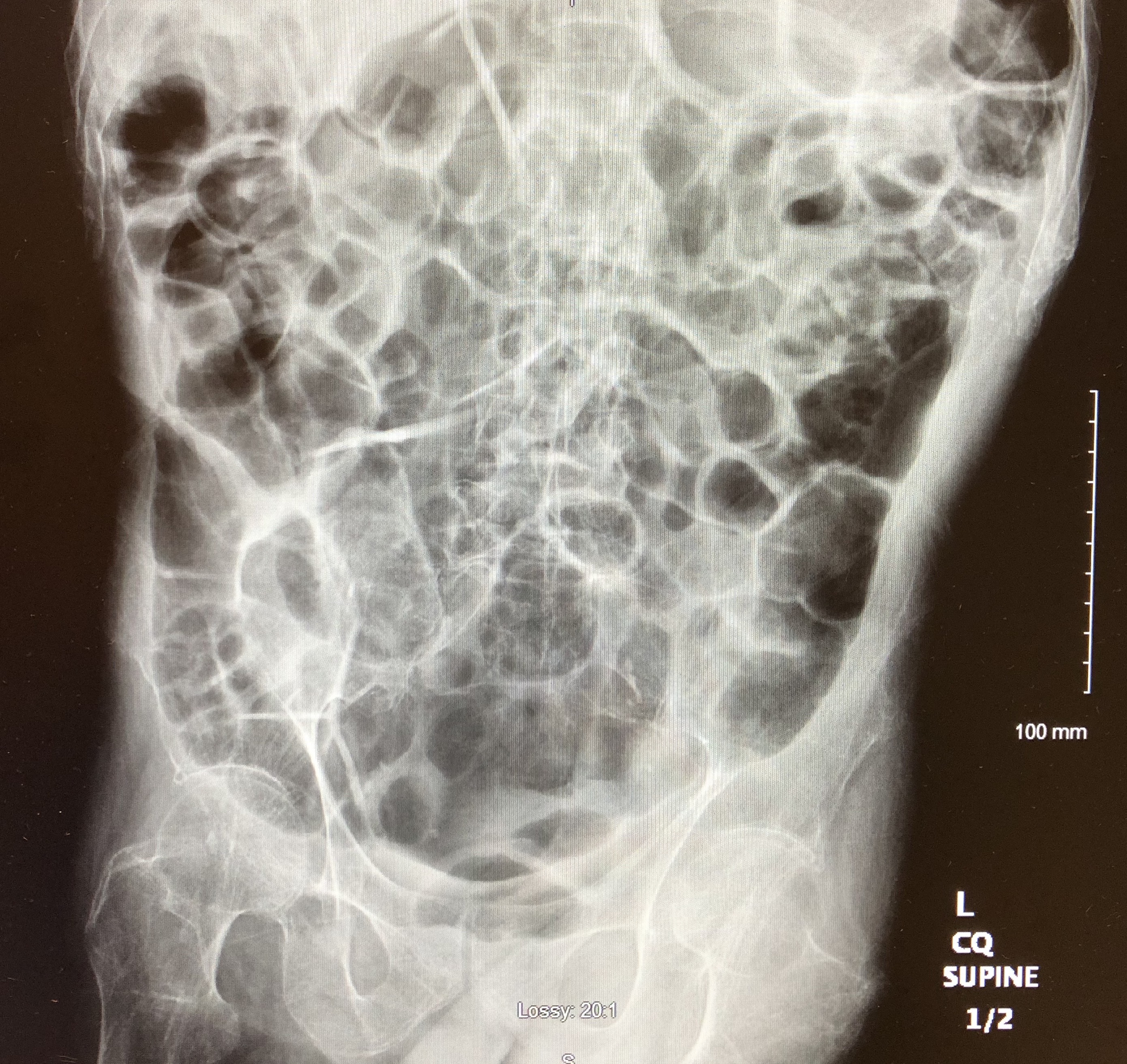

Supplement: Supplementary file 4 [file jetem-5-4-v19-supp4.jpg]
